# Molecular dynamics simulations elucidate oligosaccharide recognition pathways by galectin-3 at atomic resolution

**DOI:** 10.1016/j.jbc.2021.101271

**Published:** 2021-10-05

**Authors:** Jaya Krishna Koneru, Suman Sinha, Jagannath Mondal

**Affiliations:** Tata Institute of Fundamental Research, Center for Interdisciplinary Sciences, Hyderabad, India

**Keywords:** galectin, oligosaccharide, recognition, MD simulation, ligand binding, Markov state model, drug residence time, CRD, carbohydrate recognition domain, CV, collective variable, ITS, implied timescale, LacNAc, N-acetyllactosamine, MD, molecular dynamics, MFPT, mean first passage time, MSM, Markov state model, tICA, time-lagged independent component analysis

## Abstract

The recognition of carbohydrates by lectins plays key roles in diverse cellular processes such as cellular adhesion, proliferation, and apoptosis, which makes it a therapeutic target of significance against cancers. One of the most functionally active lectins, galectin-3 is distinctively known for its specific binding affinity toward β-galactoside. However, despite the prevalence of high-resolution crystallographic structures, the mechanistic basis and more significantly, the dynamic process underlying carbohydrate recognition by galectin-3 are currently elusive. To this end, we employed extensive Molecular Dynamics simulations to unravel the complete binding event of human galectin-3 with its native natural ligand N-acetyllactosamine (LacNAc) at atomic precision. The simulation trajectory demonstrates that the oligosaccharide diffuses around the protein and eventually identifies and binds to the biologically designated binding site of galectin-3 in real time. The simulated bound pose correlates with the crystallographic pose with atomic-level accuracy and recapitulates the signature stabilizing galectin-3/oligosaccharide interactions. The recognition pathway also reveals a set of transient non-native ligand poses in its course to the receptor. Interestingly, kinetic analysis in combination with a residue-level picture revealed that the key to the efficacy of a more active structural variant of the LacNAc lay in the ligand’s resilience against disassociation from galectin-3. By catching the ligand in the act of finding its target, our investigations elucidate the detailed recognition mechanism of the carbohydrate-binding domain of galectin-3 and underscore the importance of ligand–target binary complex residence time in understanding the structure–activity relationship of cognate ligands.

Apart from being the key energy source to human body, carbohydrates are involved in many extra- and intracellular functions such as cell adhesion, cell recognition, known to be informational encoders in cell signaling pathways. Their biological activities are usually mediated by carbohydrate recognizing proteins, such as lectins (1, 2). Galectins are a family of animal lectin receptors, which show high affinity to β-galactosides (3). Till now 15 types of galectins have been identified in the mammals. At least extracellularly, galectins generally function by binding to the carbohydrate portion of glycoconjugates on the cell surface. In regard to this, as a family of galactoside-binding proteins, galectins have been implicated in multiple biological activities including regulation of apoptosis, cell adhesion (4) and cell signaling (5).

Recent investigations into their molecular mechanisms have been triggered by the reports of functional galectin-8 ligand specificities regulating its cell sorting (6) and by the discovery that galectin-3 induces lattice formation with branched Nglycans on cell surfaces *via* cross-linking glycosylated ligands to regulate cell surface receptor trafficking (7, 8). Several of these discoveries implicate galectins as potential targets for novel anticancer and anti-inflammatory compounds *via* inhibiting their inherent galactoside binding. As a result, last decades have seen the emergence of focused efforts on the development of high-affinity inhibitors showing selectivity for individual members of galectin. In particular, galectin-3 has remained the central protein in the midst of inhibitor discovery, as its overexpression has been associated with cancer drug resistance (9, 10), and hence it has been identified as a valuable therapeutic target in the fight against cancers (11). The majority of ongoing search for potent galectin-3 inhibitor has been invested on synthesizing novel derivatives of N-Acetyllactosamine (LacNAc), which is characterized as the prototypical natural ligand for the carbohydrate recognition domain (CRD) of galectin-3 till date. Specifically, the efforts by Nilsson group have seen the synthesis of LacNAc derivative such as methyl 2-acetamido-2-deoxy-4-O-(3-deoxy-3-[4-methoxy-2,3,5,6-tetrafluorobenzamido]-beta-D-galactopyranose)-beta-Dglucopyranoside (here by referred as “LacNAc derivative”) (12), which has been established as a high-affinity inhibitor of galectin-3. Figure 1 depicts the crystallographic structure of the galectin-3 in its apo-form and the chemical structures of the two ligands of our interest. The targets of the present work are twofold: (a) to elucidate a real-time account of the dynamical ligand recognition pathway of the native ligand by galectin-3 and (b) to establish a kinetic basis for the structure–activity relationship among related inhibitors targetting galectin-3.

**Figure 1:**
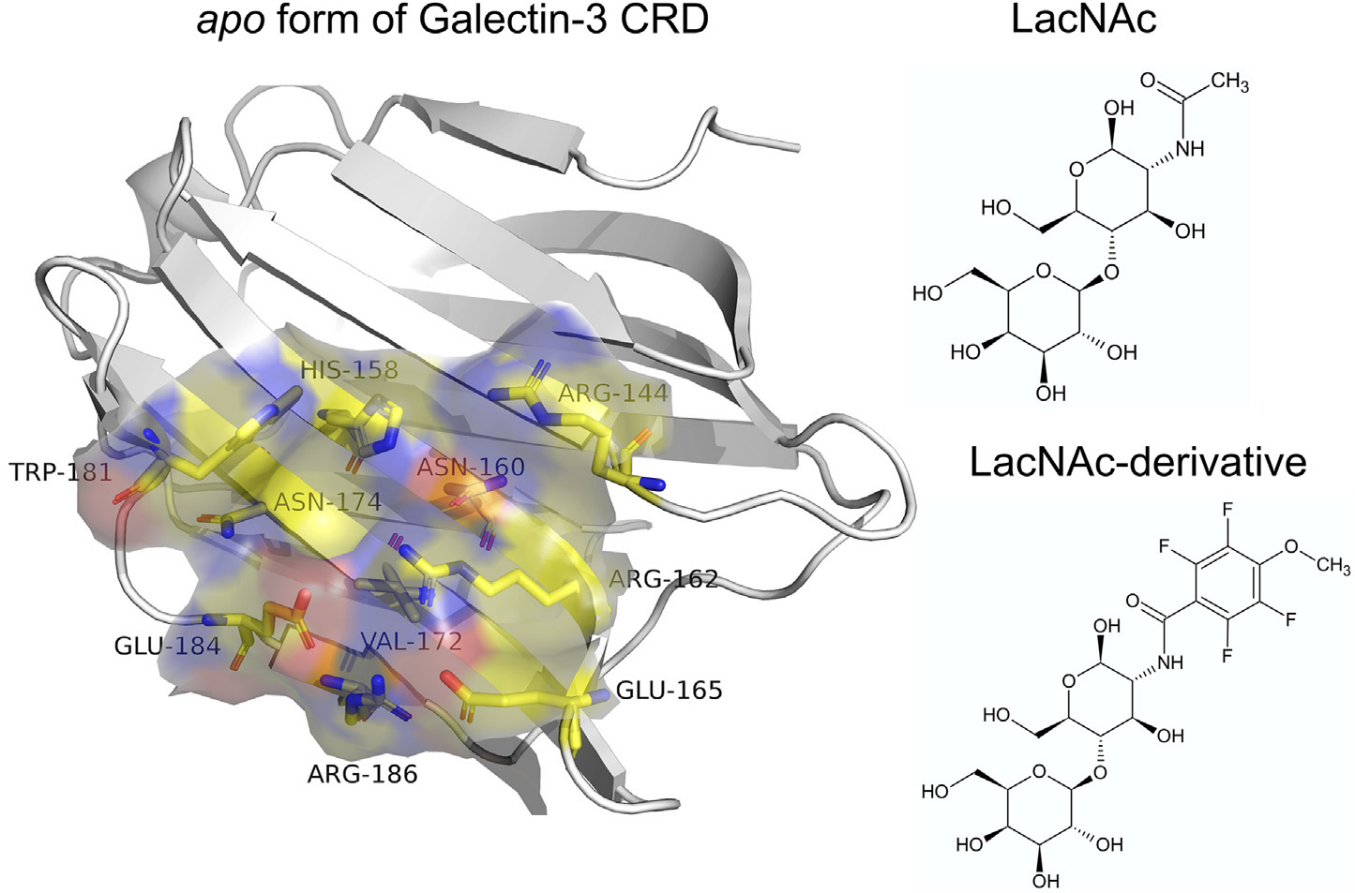
**System of interest of current investigations:***Left*, crystallographic pose of active site of galectin-3 in its apo-form. *Right*, the chemical structures of two ligands of interest, namely LacNAc and its synthetic derivative.

The crystallographic structures of human galectin-3 with bound LacNAc (see Fig. 2*A*) and “LacNAc Derivative” (see Fig. 2*B*) show a β-sandwich fold with a six-strand sheet (named S1–S6) constituting so-called “S-side” and a five-strand sheet (named F1–F5) termed as “F-side.” The structure includes a conserved shallow binding groove on the S-side over strands S4 to S6 that interacts mainly with the galactose moiety of LacNAc (13). When ligand binds within this site, key residue–ligand interactions are known to stabilize the bound pose. As most of the galectin-3 inhibitors are based on the carbohydrate skeleton, it is quite imperative to understand the mechanism of recognition process and key interactions driving the binding event. Additionally, ligand binding to a protein often comprises of formation of non-native encounter complex, which might contribute to the landscape of entire ligand-binding phenomena. In the case of galectins, as a matter of fact, these questions were never answered concretely in an atomic level so far.

**Figure 2:**
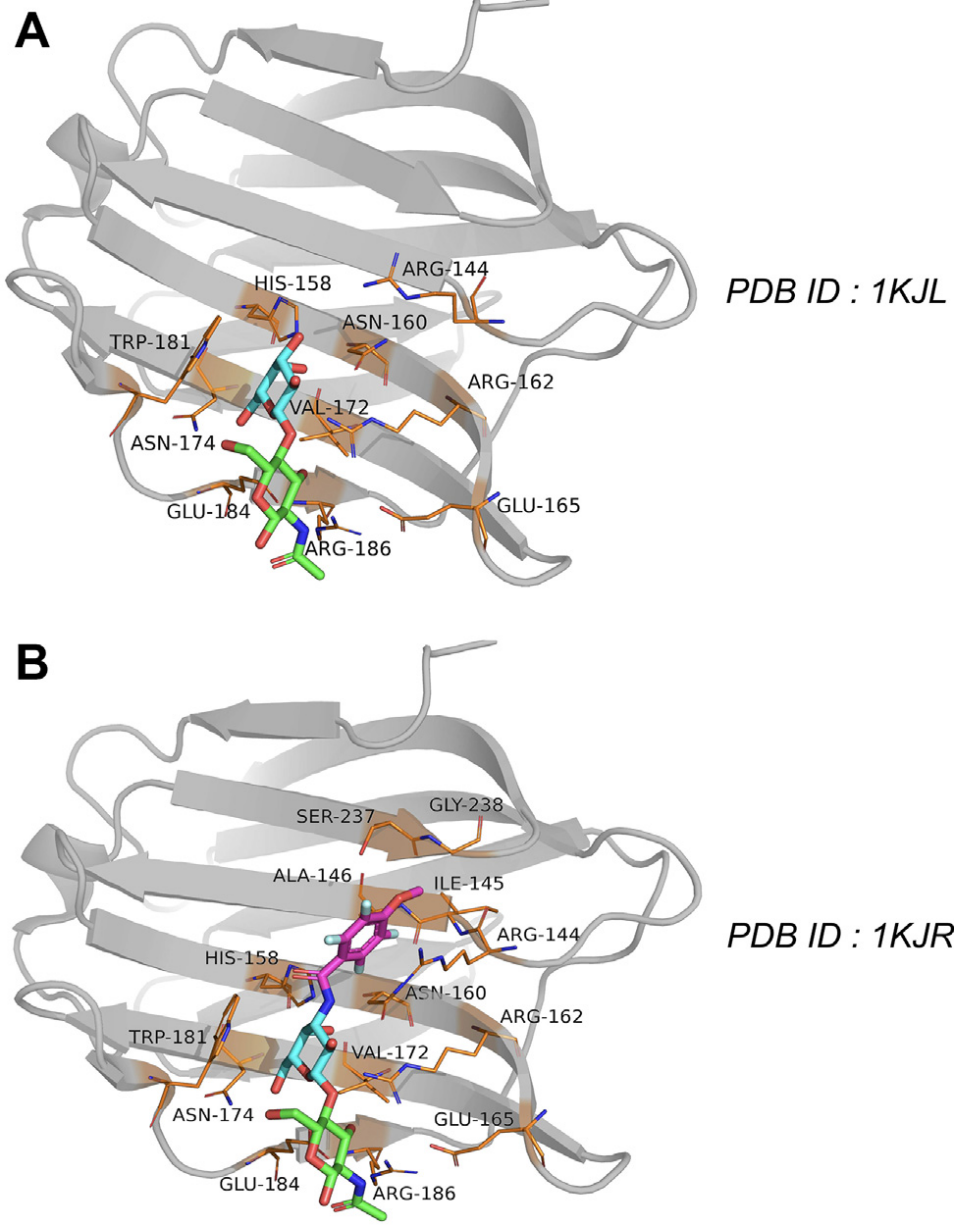
**Crystallographic pose ofgalectin-3bound withligands.***A*, ligand LacNAc (PDB: 1KJL) and (*B*) “LacNAc derivative” (PDB: 1KJR). The ligands are shown in Licorice representation. Also shown are the key amino-acid residues interacting with the ligand (*yellow stick* representation). The protein is shown in *gray cartoon* representation.

A more important purpose of the current work is to rationalize the high binding affinity of “LacNAc derivative” (12) over its parent molecule LacNAc toward galectin-3 *via* structure–activity relationship. Seminal works by Copeland *et al.* had earlier demonstrated drug residence times inside the protein active site as the true metric of drug efficacy (14, 15). The formation and duration of binary receptor–ligand complexes are elemental to many physiological processes, more so especially during drug design initiatives. The overall structure–activity relationship is generally quantified using binding parameters such as IC50 or *K*_*d*_. However, as an emerging theme, drug–target binary complex residence time has been debated as an alternative perspective on lead optimization. The dissociative half-life of the receptor–ligand binary complex has been identified as a crucial metric of compound optimization especially during hit to lead calculations. Multiple investigations have shown evidence that long residence time has specific advantages such as duration of pharmacological effect and selectivity of protein target (14, 15). This hypothesis, together with the requirement of ligand’s thermodynamic stability at binding pocket, has worked as a basis of the current work.

In light of these questions pertaining to interaction of galectins with a carbohydrate-based ligand, we employ classical molecular dynamics (MD) simulation to elucidate the mechanism of binding of prototypical ligand LacNAc and one of its synthetic derivatives with the CRD of galectin-3. Specifically, going beyond the tradition of *in silico* docking approaches, in this work, we simulate complete kinetic process of spontaneous binding of both the ligands into CRD of galectin-3. The simulations vividly capture the event of spontaneous binding of both the ligands, from solvent to galectin-3 pocket, at an atomic precision. The eventual simulated bound pose is in excellent agreement with crystallographic one. The simulations, together with the Markov state model (MSM) (16, 17, 18, 19) analysis of the aggregated trajectories, elucidate kinetically feasible pathways of ligand recognitions in both systems and provide key insights into binding mechanisms and key non-native intermediates. An analysis of the ligand-binding/unbinding kinetics points out that the origin of experimentally reported large difference in the binding-affinity between the two ligands lies in their different ligand-residence time. A residue-level analysis identifies key interactions of the binding pocket residues (Trp181, Arg144, and Arg162) with the tetrafluorophenyl ring of the derivative as the key determinant for the synthetic ligand to latch into the pocket.

## Results

### Simulated trajectory captures the ligand-recognition event by galectin-3

**Figure 3:**
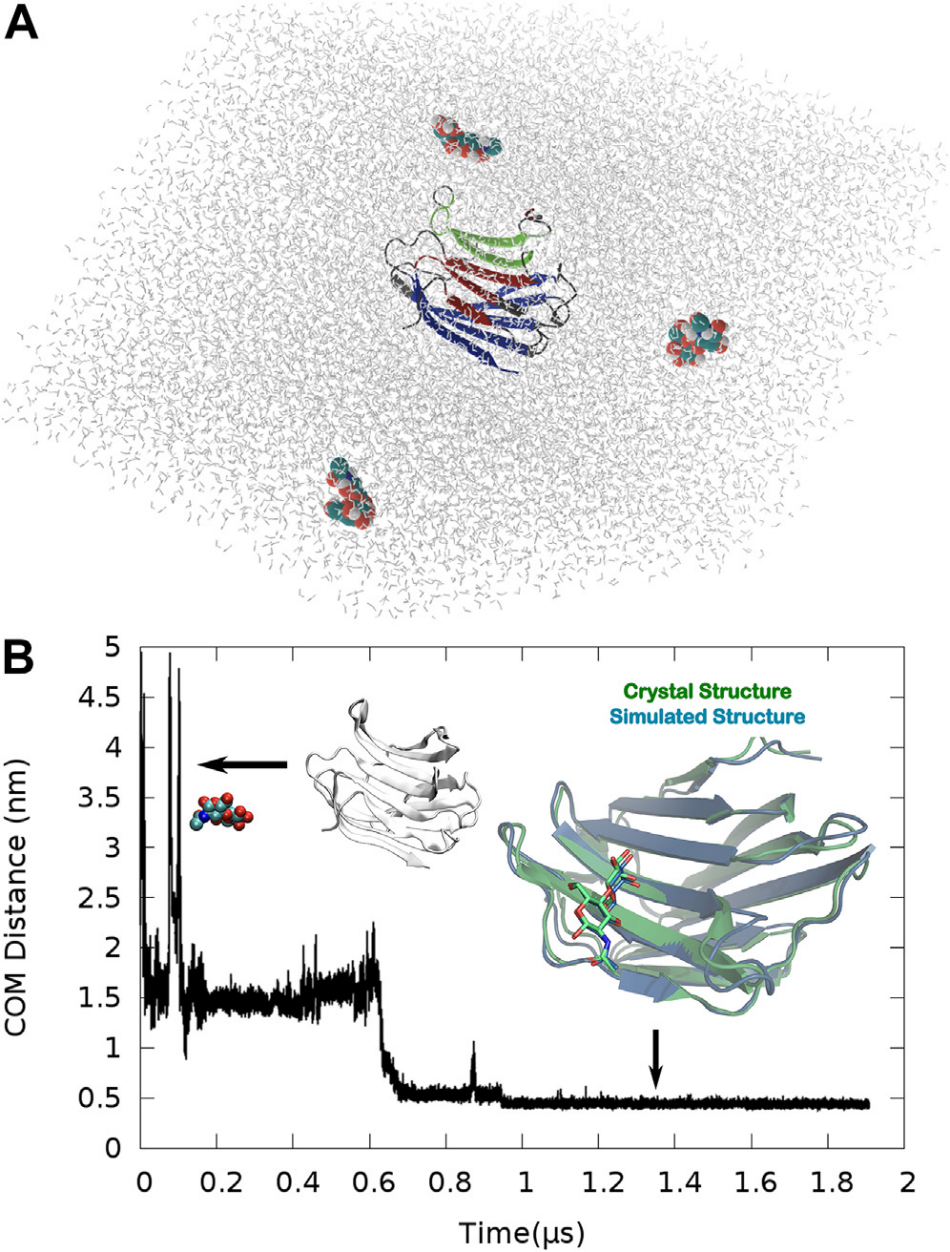
**Simulation captures crystallographic pose.***A*, current simulation setup with initial configuration of galectin-3 in the presence of LacNAc molecules in aqueous media. *B*, the time profile of distance between LacNAc and the designated binding pocket of the Galectin. Also annotated are the representative snapshots of ligand-unbound and bound pose (overplayed with the crystallographic pose (pdb id: 1KJL)).

Notwithstanding the prevalence of static crystallographic poses of carbohydrate-binding domain of galectin-3 in its ligand-bound pose (12), a dynamical account of the oligosaccharide-recognition process by the protein is currently elusive. In a bid to explore the mechanism of the recognition process of native ligand LacNac by galectin-3, we undertook an ambitious initiative of MD simulations to see if we can mimic the full process of ligand recognition: in other words, if the ligand can be individually caught in its act of binding to its designated cavity within the simulation timescale. Accordingly, we initiated multiple long independent unbiased all-atom MD simulations to explore the diffusion of multiple copies of ligand copies, initially randomly distributed in water, around galectin-3 (see Experimental procedures and model and Fig. 3*A* for simulation setup).

Movie S1 depicts one such representative MD trajectory of a single LacNAc molecule in its course to bind the CRD domain of galectin-3. The movie demonstrates that LacNAc initially freely diffused in solvent media and occasionally interacted with various part of the protein. Eventually, the ligand identified the S-side of the protein and settled in the designated CRD of the protein within a microsecond-long simulation period. The overlay (Fig. 3*B*) of the final LacNAc-bound pose (green), as obtained at the end of the simulation, with that of crystallographic pose (pdb id: 1KJL) (gray) shows near-identical match, thereby attesting to the successful capture of the bound pose in computer simulation. The visual inspection indicated that the final simulated pose recapitulates all key interactions with multiple amino acid residues identical to crystallographic structures. The time evolution of distance between LacNAc and binding pocket quantifies successful ligand-binding process as the distance decreases and eventually gets plateaued at an average value of 0.4 nm (Fig. 3, bottom).

### Identification of key residues stabilizing the ligand in the galectin-3-binding site

**Figure 4:**
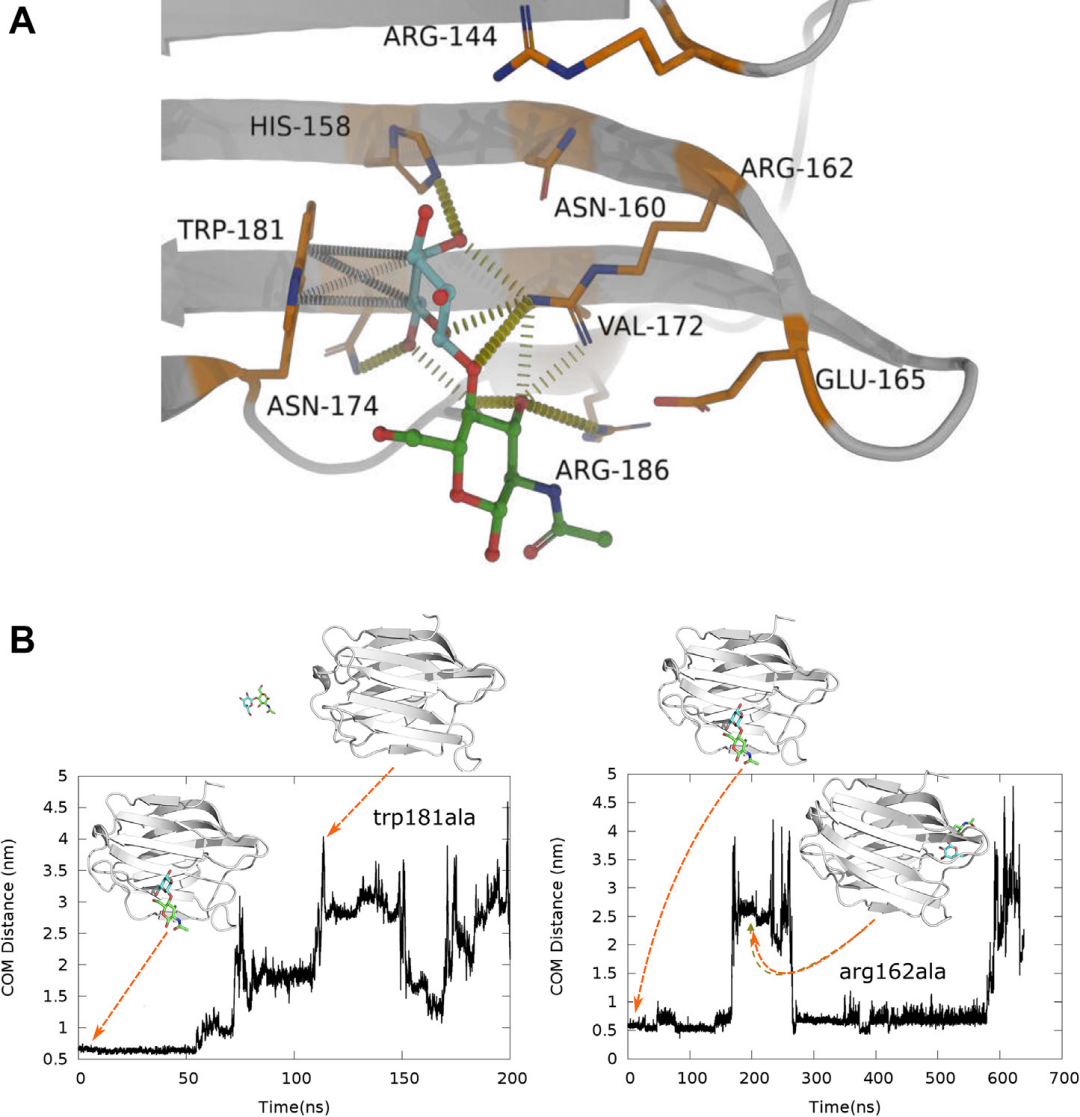
**Assessing residues crucial for ligand binding.***A*, closed view image of the binding pocket residues interacting with LacNAc in its native bound pose. *B*, assessing the stabilizing effect of key residues around bound pose *via in silico* mutation: Time profile of pocket–ligand separation in simulations subsequent to Trp181Ala and Arg162Ala mutation in ligand-bound pose.

The expanded snapshot in Figure 4*A* illustrates the key interactions between the LacNAc and the aforementioned binding pocket residues at the completion of binding event. In particular, the ligand is found to form direct hydrogen bonds with asn174, glu184, arg186, and arg162. Water-mediated hydrogen bonds are also found with the LacNAc through asn160 and glu165, which form a stabilizing network of interactions. We have also observed a charge center-based electrostatic interaction of the ligand with arg162. These are interestingly the same residues identified in NMR and X-ray crystallographic studies as the signature interactions between LacNAc and residues in binding site (13). The time profiles depicted in Fig. S1 show the formation of consistent hydrogen bonds between several residues and LacNAc after it settles into the binding pocket. Along with the electrostatic interactions, we also find strong stacking interaction between Trp181 and hydrophobic atoms of LacNAc. This is consistent with previous report that Trp181 acts as a receptor for galactose residues in polysaccharides during binding *via* cation–π hydrophobic interaction, which play a main role in the binding affinity of polysaccharides with the protein (12). Overall, the visual inspection of our simulation trajectories and subsequent analysis confirm the presence of these signature stabilizing interactions in the ligand-bound pose.

Our analysis points toward two key residues (Trp181, Arg162), which are instrumental in this particular molecular recognition event. To further assess the importance of these residues in stabilizing the ligand-bound pose, we individually mutated two aforementioned key residues Trp181 and Arg162 to alanine in the LacNAc-bound pose *via in silico* approach and subsequently performed independent control simulations with the *in silico* mutated protein. Figure 4*B* depicts the simulated pocket–ligand distance profiles after the *in silico* Trp181Ala and Arg162Ala mutation in the ligand-bound pose. We find that both the mutations orchestrate the unbinding of the ligand from the designated cavity within a short timescale. Together, these observations from control simulations lend credence to a significant role of these key residues in stabilizing the ligand-bound pose. Interestingly, we find that the overall structure of the binding pocket of galectin-3 remains mostly unchanged during the period of simulation, which spanned both the event in which LacNAc had explored solvent media and it remained bound to the CRD domain. This is reflected well in mostly unchanged root-mean-squared deviation (RMSD) profile of galectin-3-binding pocket throughout the simulation trajectory (see Fig. S2). This observation is consistent with prior report (20) of a stable binding pocket of galectin in both apo and ligand-bound form.

### Simulation identifies metastable non-native encounter complex

**Figure 5:**
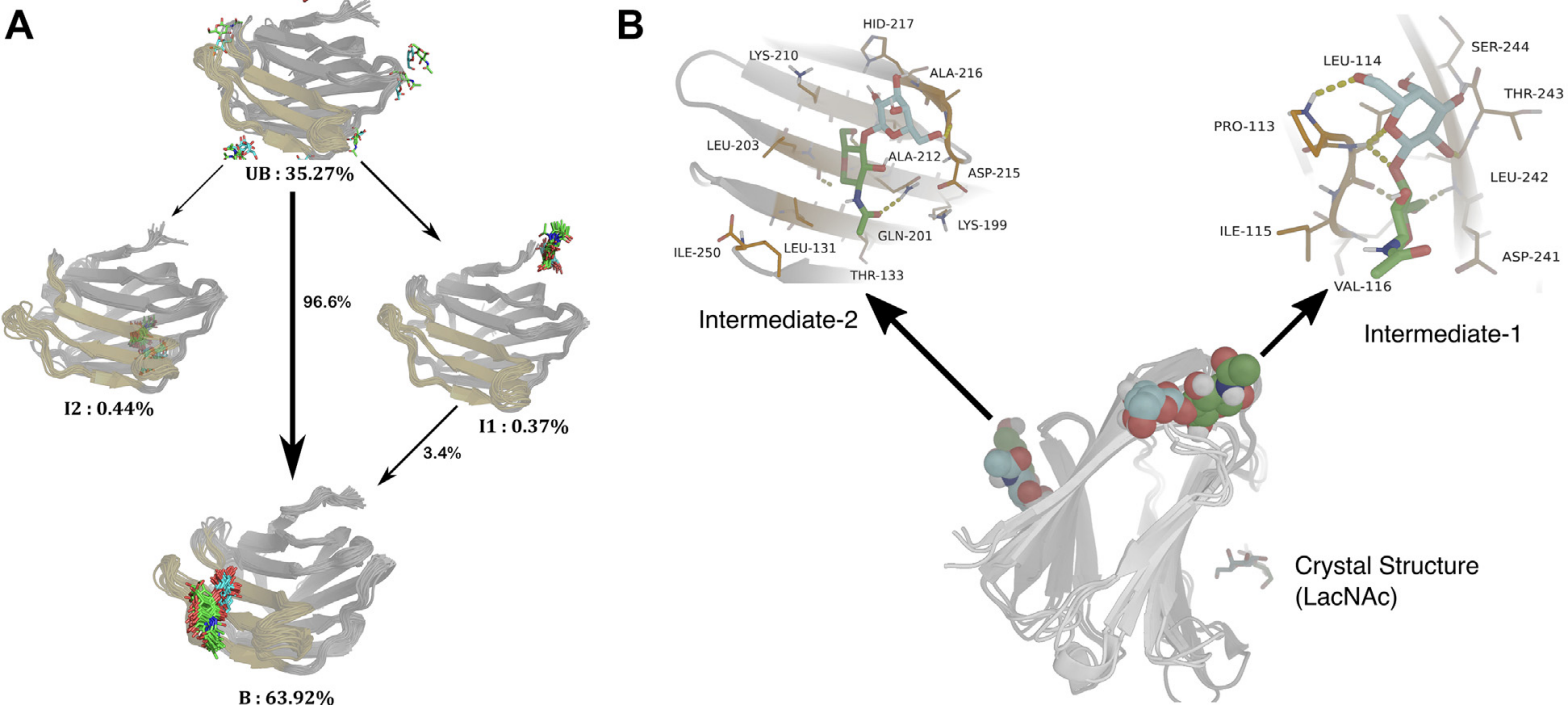
**A Markov state model characterizing galectin-3 recognition of LacNAc.***A*, network showing the LacNAc-binding pathway. Path flux is represented with *thickness of arrows* and path percentages are indicated. *B*, key interactions stabilizing transient intermediate I1 and I2 of LacNAc.

For a quantitative understanding of the kinetics and thermodynamics of the binding event of the native ligand LacNAc, we curated all simulation trajectories into a comprehensive statistical model, known as Markov state model (MSM) (16, 17, 18, 19) (see Experimental procedures and model). As shown in Figure 5*A*, the analysis predicted the existence of LacNAc in four different sites around the protein: Visual inspection and overlay with the crystallographic pose identified states U and B as the solvated and native-bound states of galectin-3 respectively, which are the key major states (see Table 1 for their relative populations). The standard free energy of binding of the parent ligand (LacNAc) computed using these equilibrium populations was $\Delta G_{sim}^{0}=-3.89\pm 0.9\;kcal.mol^{-1}$, which is in reasonable agreement with experimental value (12) of $\text{Δ}G_{exp}^{0}=-5.25\;kcal.mol^{-1}$.

**Table 1:** Characteristics of the all four macrostates for galectin-3/LacNAc system

Macrostate	Stationary population	Committor probability
U (unbound)	35.27%	0
I1	0.37%	0.017
I2	0.44%	0.016
B (bound)	63.92%	1.0

Apart from the fully unbound and native bound pose of LacNAc, our quantitative model also predicts the occurence of a pair of short-lived metastable protein–ligand encounter complexes, labeled here as I1 and I2. (Fig. 5, *A* and *B*) The visual inspection of ligand locations in these two non-native encounter complexes (see Fig. 5*B* for an expanded view of I1 and I2) shows that LacNAc is present either in a distal location (state I1) or in the reverse side (the F side) of the CRD (state I2). A kinetic analysis (see Experimental procedures and reference (21)) elucidates all probable pathways that LacNAc would utilize for traversing from fully unbound (U) state to native bound (B) pose. Figure 5*A* represents the network of transition paths of LacNAc with their respective net-flux contribution. Interestingly, as shown in Figure 5*A*, we find that overall binding process of LacNAc is mostly dominated by the direct U→B pathway On the other hand, only a minor contribution to the overall pathway comes *via* U→I1→B transition. Interestingly, any pathway through I2 does not contribute to the ligand binding, which is mostly due to the fact that the ligand is located on the reverse side of the CRD in this non-native pose, suggesting it to be a “off-pathway” intermediate complex.

What is the origin of these intermediates? Figure 5*B* highlights the key interactions stabilizing LacNAc in I1 and I2 intermediate. The ligand is found to bind in I1 site, stabilized through backbone hydrogen bonds. On the other hand, the major contribution comes from side chains of asp215 and gln201, although gln201 main chain also forms a hydrogen bond with the ligand. In the I1 site, the main-chain hydrogen bonds are mostly contributed by thr243, leu242, pro113, and leu114. The methyl group from acetamido functional group of the ligand is found to have hydrophobic contacts with 1le115 and val116. It is most probably the absence of strong hydrogen bonds between ligand and side chains of pocket residues, which makes the stability of the poses I1 and I2 comparatively weaker than the crystallographic binding pocket, which eventually makes these intermediates a transient resting pocket, before it finally binds to the experimentally defined binding pocket. Interestingly, previous NMR investigation of binding of long-chain carbohydrate-based ligand, for example, galactomannans to galectin-3 (22) had shown that F-side of the protein exhibits significant binding affinity toward long-chain carbohydrates. From the aforementioned interactions between ligand and residues in I1 and I2, as presented in the previous report of flexible conformational behavior of polysaccharides in solution environment (22), we can conjecture that the metastable states, which are obtained from the simulation trajectories, might assist CRD in binding with long polysaccharides and the aforementioned residues might potentially be a subset of the residues, which might help in affinity toward carbohydrates.

### Exploring common traits of galectin recognition in a rationally designed galectin inhibitor

While LacNAc is the prototypical ligand extensively investigated as a reference for binding to galectin-3, a key thrust in medicinal chemistry involving galectin-3 has been the rational design of competitive inhibitors. A key question is: how conserved is the overall galectin-recognition mechanism, as elucidated in the current investigation for parent ligand LacNAc? In this regard, we chose a “LacNAc derivative” (see Fig. 1), which has been rationally designed and crystallized by Nilsson *et al.* (see Fig. 2). This derivative has shown more superior biological activity than LacNAc against galectin-3, and hence it gives us the opportunity to explore the commonalities in its features of the binding mechanism with the parent ligand, as well as to establish a structure–activity relationship between these two ligands.

We exploited a similar simulation strategy for “LacNAc derivative” (LacNAc-2,3,5,6-tetrafluoro-4-methoxy-benzamide), as described previously in the case of LacNAc, to discover the traits of its galectin-3-binding process. Accordingly, we carried out a similar set of long MD simulations to see if the trajectory of LacNAc derivative can be derived in its act of getting bound to galectin-3. Very interestingly, during the time course of the long simulation, we find that, similar to its parent ligand, the LacNAc derivative also settles in the designated binding pocket, with accurate recapitulation of the crystallographic bound pose (see Fig. S3). A comparison of pocket–ligand distance profiles of LacNAc and its synthetic derivative (see Fig. S3) indicates that overall binding timescales are very similar for both the ligands, suggesting that the estimated on-rate constants of both the ligand would be of same order of magnitudes. As would be divulged later, this would be eventually confirmed by an estimate of these kinetic constants.

**Figure 6:**
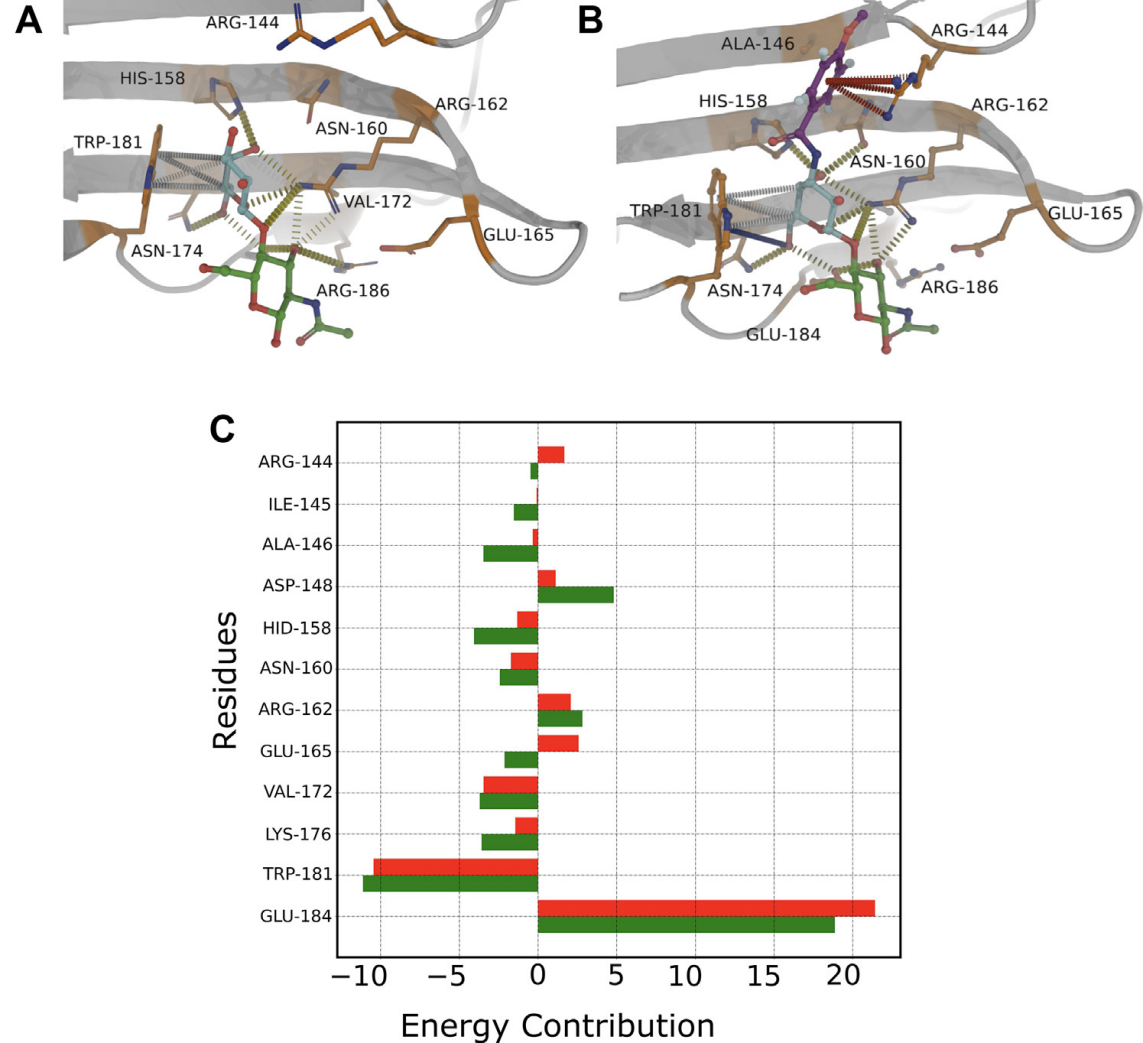
**Molecular determinants ofgalectin-3/ligandrecognition.***A*, key ligand–residue interactions stabilizing the bound pose. Hydrogen-bond formation between His158, Arg162, Asn164, and Glu184 with ligand after the binding event and stacking overlap between aromatic side chain of Trp181 and galactose residue of ligand. *B*, the interaction diagram for LacNAC derivative. *C*, MMPBSA per-residue energy contributions. The *red bars* correspond to LacNAc while the *green bar* corresponds to the derivative.

Figure 6 compares the interaction between the binding pocket residues and LacNAc derivative with that with the native ligand. While the derivative retains most of the interaction with the binding pocket residues as in native ligand LacNAc, it also primarily establishes contact with Arg144 through pi–cation and donor–pi interactions due to the presence of an extra 4-F ring (See Fig. 6*B*). An additional hydrogen bond through the methoxy group has also been noticed with arg144. We also find carbon–pi interaction between trp181 and hydrophobic atoms of galactosamine ring. Besides the stabilizing interactions of the 4F-ring, multiple hydrogen bonds are observed to be formed during the ligand arrest into the galectin-3-binding pocket, most notable among those are with arg162, glu184, and asn174, all of which are contributed from galactosamine ring. We have conducted residue-wise free-energy analysis to understand the relative importance of residues in the ligand-binding pocket for both the ligands (Fig. 6*C*, red bar for parent ligand, green bar for the derivative). Most of the residues in the binding pocket contribute favorably toward binding of LacNAC derivative over the parent ligand. Notable among them are arg144, his158, asn160, arg162, trp181, and glu184. In particular, arg144 shows favorable interaction with the derivative, whereas it is noted that it has no binding contributions for the parent ligand.

A similar quantitative analysis for LacNAc derivative suggests that its protein-recognition kinetics can be adequately described by three key ligand states at different parts of protein or solvent (Fig. S4). We find that, similar to its parent ligand LacNAc, the ligand-binding equilibrium in the derivative is also majorly guided by direct transition between ligand-unbound state of the proteins (UB) to its crystallographic bound pose (B). Nonetheless, the analysis also predicts the presence of a single transient non-native encounter complex (I) with a very low population, and there is a small but finite probability of an alternate pathway in which the ligand-binding process can be mediated by this short-lived intermediate (see Table 2). A visual comparison between this transient intermediate (I) of the LacNAc derivative, with those of the LacNAC (*i.e.*, I1 and I2) suggests that the I2 resembles that of the transient intermediate (I) observed in case of the derivative. A closer observation of the snapshot (see Fig. S4) suggests that transient intermediate of LacNAc derivative is located on the same place of protein as in I2, making hydrogen bonds with gln201, gln220, lys210, and his217. Additionally, the intermediate corresponding to the derivative makes pi–carbon interaction with leu203 and makes both donor–pi and pi–carbon interactions with his217. We speculate that these additional interactions make for relatively higher population (1.97%) of the intermediate (I) macrostate of LacNAc derivative than its corresponding counterpart (intermediate I2) of parent ligand LacNAc (0.44%).

**Table 2:** Characteristics of the MSM-derived macrostates for galectin-3/LacNAC-derivative system

Macrostate	Stationary population	Committor probability
U (unbound)	3.27%	0
I	1.97%	0.315
B (bound)	94.80%	1.0

### Longer ligand residence time holds the key to ligand efficiency in galectin-3

Irrespective of the similar signatures of the binding events by both the ligands, it is intriguing to note the report of superior binding affinity of the LacNAc derivative over the native ligand (12). To decipher the origin of the superior efficiency of the LacNAc derivative over the native ligand, we explored the free energetics and kinetics (see description in Experimental procedures).

Interestingly, an estimation of standard binding affinity of the derivative, based on the relative population of ligand-bound and -unbound macro state, yielded a value of $\Delta G_{sim}^{0}=-5.70\pm 0.8\;kcal.mol^{-1}$. A comparison of computed binding free energies (see Table 3) between two ligands ($\text{Δ}G_{sim}^{0}=-5.70\pm 0.8\;kcal.mol^{-1}$ for *versus* −3.8 ± 0.9 *kcal*.*mol*^−1^ for LacNAc) confirms the superior binding affinity of LacNAc derivative over LacNAc toward galectin-3, in accordance with the experiments.

**Table 3:** Comparison of computed binding constants across two ligands

Ligand/protein system	$\Delta G_{sim}^{0}\left(Kcal/mol\right)$	*K*_*on*_ (*M*^−1^*s*^−1^)	*K*_*off*_ (*s*^−1^)
LacNAc/Galectin	−3.8	2.8 × 10^8^	1 × 10^6^
LacNAc derivative/Galectin	−5.70	1.4 × 10^8^	0.038 × 10^6^

The kinetic aspect of ligand recognition process involves a delicate balance between binding or on-rate constant (*K*_*on*_) *versus* unbinding or off-rate constant (*K*_*off*_). In order to delve deeper into the origin of superior binding affinity of LacNAc derivative over the parent ligand, we computed the plausible rate constants for transition from the ligand-unbound macrostate to the ligand-bound macro state and vice versa (see Experimental procedures for equations of rate constants), following the protocol of our past investigations (23, 24). The ligand binding on-rate constant (*K*_*on*_) for LacNAc, as derived here, is estimated to be 2.8 × 10^8^ *M*^−1^*s*^−1^, while for its synthetic derivative, a similar analysis of *K*_*on*_ yields a value of 1.4 × 10^8^ *M*^−1^*s*^−1^ (see Table 3). This indicates very similar order of magnitude of ligand-binding rate constants for both the ligands, while suggesting a slightly faster binding kinetics for the natural ligand LacNAc than that of its derivative.

However, on the other hand, binding off-rate constant (*K*_*off*_) was estimated to be 1 × 10^6^ *s*^−1^ for LacNAc and 0.038 × 10^6^ *s*^−1^ for its derivative, indicating around 25 times slower unbinding rate constant in LacNAc derivative than the parent ligand. Since the off-rate constant is inversely related to the ligand-residence time in the pocket, the analysis also indicates that LacNAc derivative would have longer residence time in the pocket than LacNAc. Together, significantly slower unbinding rate of LacNAc derivative from the pocket of galectin-3 than that of LacNAc, coupled with very similar on-rate constants (see Table 3) in both cases, dictates that off-rate constant would be the key determinant of the superior binding efficiency of the synthetic derivative over the parent ligand LacNAc. Our observation of longer ligand-residence time or slower off-rate constants guiding ligand efficiency in galectin-3 echoes past hypothesis of Copeland *et al.* (14, 15) of off-rate constant as a key determinant of ligand efficiency. This concept was primarily observed from studies of mutation-based resistance to inhibitors of HIV-1 protease by Maschera *et al.* (25), who studied mutants of the HIV-1 protease, which were resistant to the AIDS drug saquinavir. Key findings from this study proposed that the *in vitro* values of inhibition constants (Ki) for the wild-type and mutant enzymes and the corresponding IC50 values for inhibition of viral replication in cell culture were both strongly correlated with the off-rate (*k*_*off*_) of the saquinavir protease complex, whereas the association rate constant (*k*_*on*_), in contrast, varied less than twofold among the mutant enzymes.

### Dissecting the molecular determinants of ligand residence

**Figure 7:**
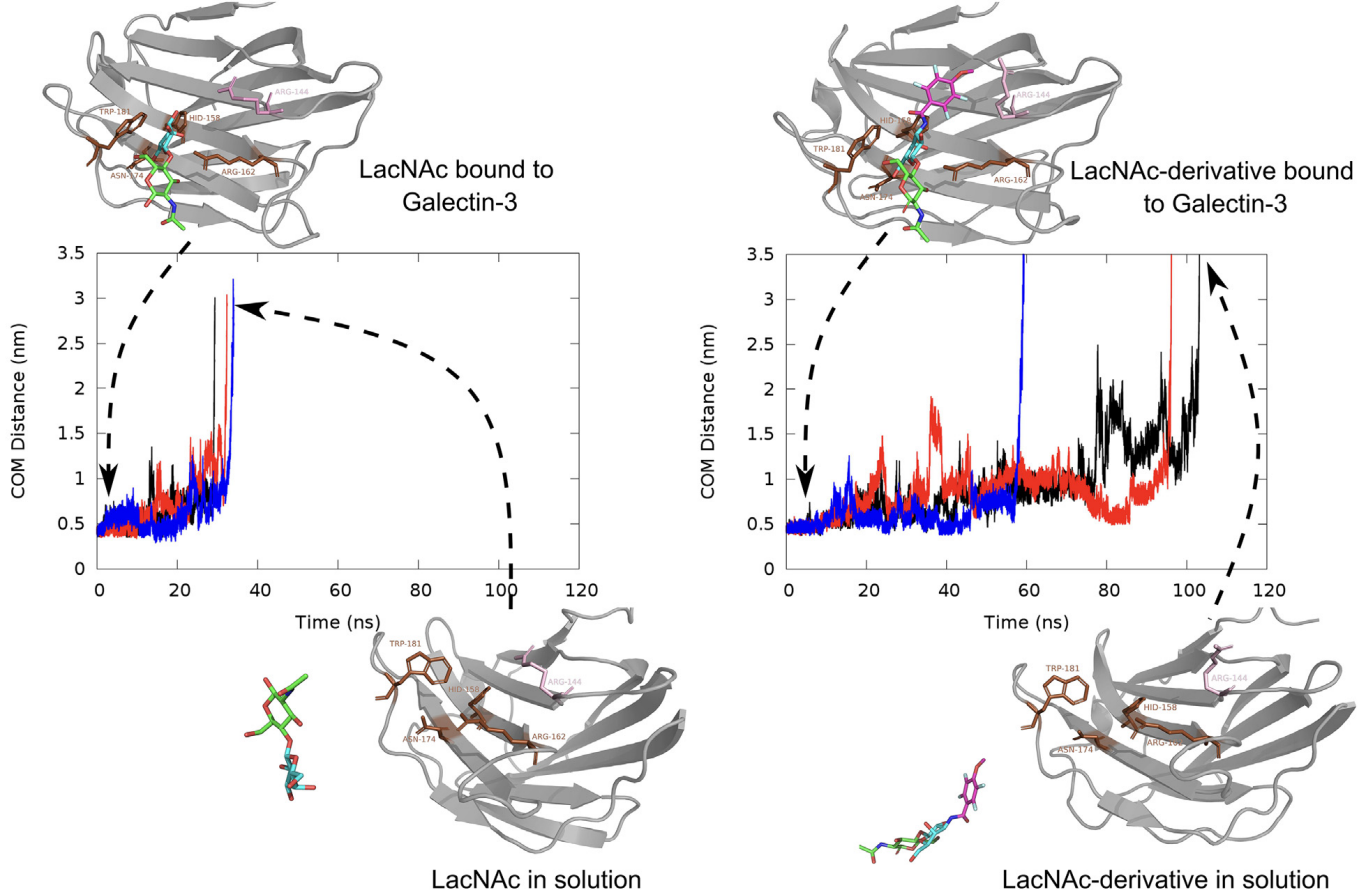
**Assessing ligand resilience in the binding pocket.** Comparison of ligand-egress trajectory as obtained from metadynamics simulations of unbinding of LacNAc and LacNAc derivative.

Nonetheless, the predicted longer residence time of the LacNAc derivative over its parent ligand in the galectin-3 pocket warrants a molecular interpretation. Toward this end, we employed a metadynamics simulation approach to explore the relative resilience of the synthetic derivative over LacNAc in the binding pocket of galectin-3. Specifically, *via* metadynamics simulation, we investigated if the egress of LacNAc derivative from the pocket would have taken relatively longer time than that of LacNac and if we can dissect the key ligand–residue interactions accounting for this difference. Figure 7 compares the ligand–pocket distance profile of LacNAc and its derivative as we simulate their unbinding process from the native bound pose of the galectin-3 *via* metadynamics simulation. Within the same metadynamics protocol and compared across multiple trajectories, we find that LacNAc derivative in general would take considerably more time for egress from the bound pose than the parent ligand.

**Figure 8:**
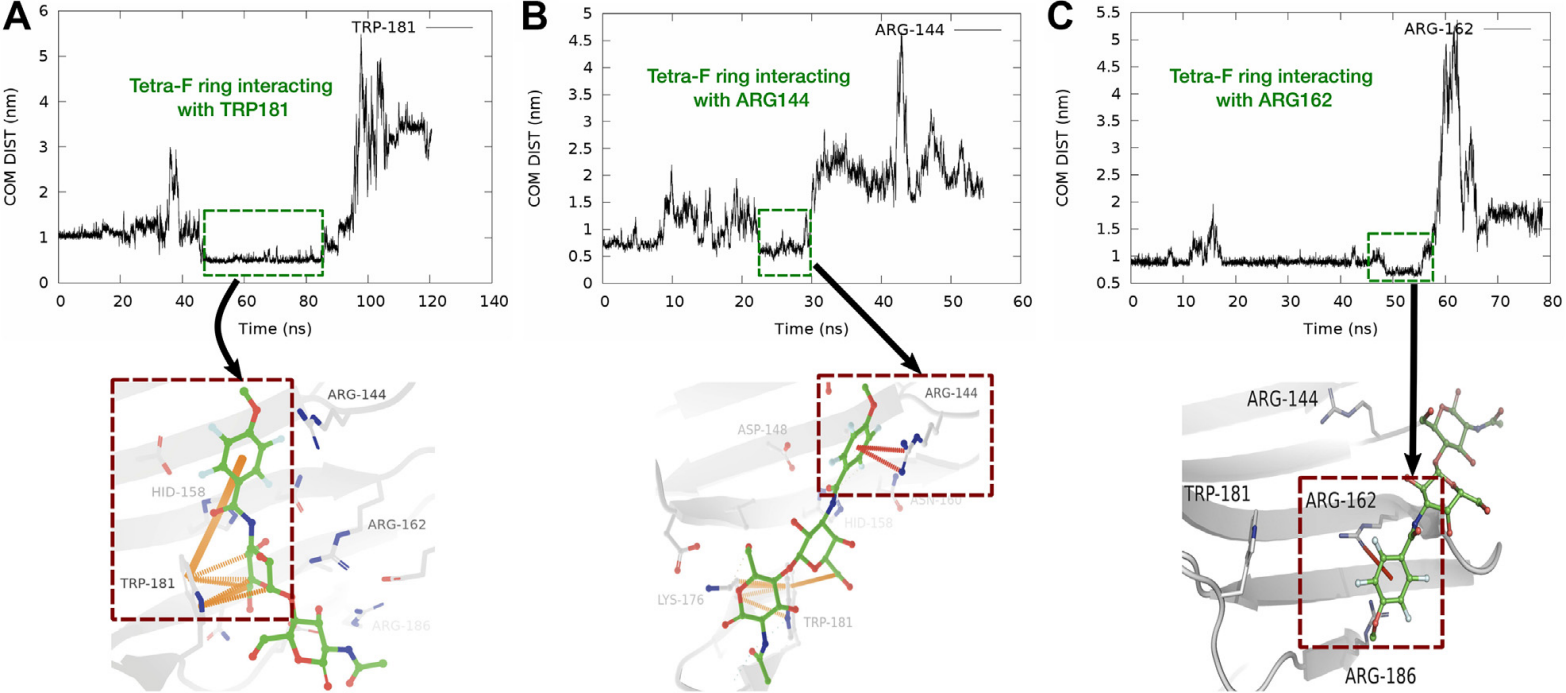
**Key interactions oftheLacNAc derivative, which holds the ligand back for longer residence time.***A*, interactions between Trp181 and the ligand, (*B*) interaction between Arg144 and the ligand, and (*C*) interaction between Arg162 and the ligand; Pi–carbon (*orange dashes*); pi–cation (*red dashes*).

The major difference between LacNAC and its derivative is the presence of an additional tetrafluoro ring in the derivative. The presence of tetrafluoro ring in the LacNAc derivative introduces additional interactions (otherwise absent in the native ligand) with certain key residues of the binding pocket, which would prevent the ligand against unbinding from the galectin-3. Figure 8 provides a pictorial view of the key lingering interactions between tetrafluoro ring LacNAc derivative and certain key residues of the protein, which would help the ligand to reside in the pocket longer than the native ligand. As illustrated by the residue–ligand distance profiles of the meta dynamics trajectory, we find that these additional interactions between the tetrafluoro ring and certain pocket residues are stable against metadynamics biasing.

Specifically, the distance profiles (see Fig. 8*A*) suggest that Trp181 and its interaction with tetrafluoro ring are crucial for the elongated ligand residence of the LacNAc derivative. Trp181, as reported in literature and as observed in our multiple repeats of atomistic simulations (also see Fig. 4), is an important residue not only for initial stabilization of the ligand in the protein pocket but also for resisting the exit. The observation of metadynamics simulation trajectory indicated that although an initial flip of tetrafluoro ring destabilized the LacNAc derivative from its initial position, the native carbon–pi interactions between the galactosamine ring and trp181 remained intact. Additionally, we have observed partial resting of the ligand through pi-stacking interactions between tetrafluoro ring and his158. His158 also established amide–ring interactions with the tetrafluoro moiety. This is a cooperative attachment and duly supported by the residue–residue pi-stacking between his158 and trp181. A similar facilitation was also observed between lys176 and trp181 (see Fig. S5) where they are interacting through pi–carbon noncovalent bonds. These two neighboring residues (his158 and trp181) lend interim stabilization against any probable position fluctuations for trp181, which in turn is utilized by the LacNAc derivative to resist egress from the protein pocket. Moreover, the tetrafluoro ring re-establishes favorable noncovalent interactions with arg144 when the rest of the ligand has lost all native contacts during the penultimate stages of exit. This additional interaction with arg144 holds the derivative in the pocket for a longer duration than the pocket. This is illustrated *via* a resilient Arg144/tetrafluoro ring interactions during the ligand egress (see distance profile in 8B). Fig. S6 renders the detailed pictorial representation of interaction between LacNAc derivative and the binding pocket residues. As shown in Fig. S6 and in the distance profile (8C), during the final stages of egress, as last resorts, the tetrafluoro ring moiety initially clings to trp181 through pi–pi stacking interactions and finally establishes pi–cation interaction with arg162, before it ultimately leaves in the bulk.

## Discussion

The mechanism of ligand recognition by biologically important receptors has remained a crucial topic of interest. Computational docking approach has remained a traditional route for exploration of the key interactions responsible for ligand/receptor complex formation, which aids experimental investigations. However, the approach has often been found to be riddled with shortcomings involving limited exploration. More often, the traditional experimental techniques also lack temporal and spatial resolution to elucidate the rare transient events in the kinetic pathways of ligand approach to the receptor. In this respect, long-timescale all-atom MD simulation of recapitulating the actual protein/ligand-binding event comes out as a method of choice. While this is known to be a tedious and time-intensive exercise, due to its “wait and watch” nature, when successful, this approach can provide a real-time account of the ligand recognition process in an unbiased manner. The current work provides one such success story by simulating the oligosaccharide recognition process by galectin-3 at crystallographic precision. This work presents a rather rare computational example of oligosaccharide-binding simulation performed in explicit water without any artificial forces applied to guide the ligand into the receptor site. The approach enables one to observe a high-resolution trajectory of the oligosaccharide binding to the galectin-3 and comments on the potential discovery and importance of noncanonical binding sites.

**Figure 9:**
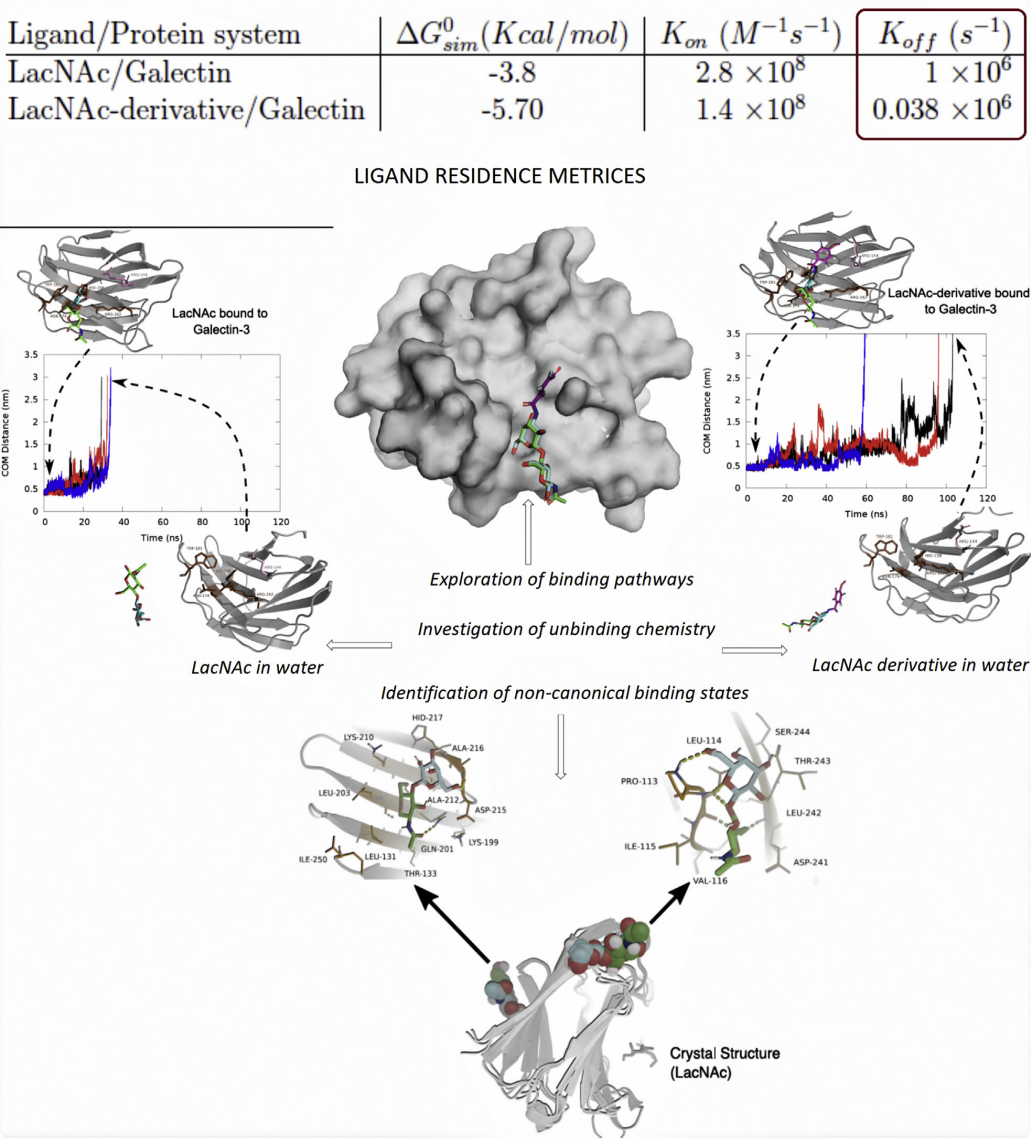
**A pictorial summary of key findings of the current simulation.** The discovery of metastable noncanonical protein/ligand encounter complex and ligand-residence time as the major factor for function of galectin-based ligand are crucial takeaway.

As summarized in Figure 9, the current work provides a glimpse of binding mechanism of galectin-3 to its native ligand LacNAc and one of its fluorine-based synthetic derivatives (referred here as “LacNAc derivative”) at an atomic precision *via* combining long unbiased binding MD simulations and statistical analysis. The simulation captures the ligands in their act of binding to its designated binding site of galectin-3. The simulated ligand-bound pose is in identical match with crystallographic structure. Among many other key stabilizing interactions, the simulation emphasizes the importance of C-H/π interaction between Trp181 and LacNAc, which is further validated by subsequent mutation-based control simulations in which the ligand was found to be dissociated in the absence of Trp residue at 181th location. The analysis of the aggregated simulation data showed the presence of transient metastable states apart from the solvated and native-bound state. However, the network of binding path, analyzed by transition path theory, predicts a direct binding from solvent to binding site as single dominant binding pathway, without any considerable net flux mediated *via* these intermediates. Overall, the estimates of relatively faster binding on-rate constant and off-rate constant of LacNAc to galectin-3, in addition to direct binding from solvent as the single dominant pathways, stand in distinct contrast to the traits of ligand-binding events in proteins with solvent-occluded binding site (23, 24).

**Figure 10:**
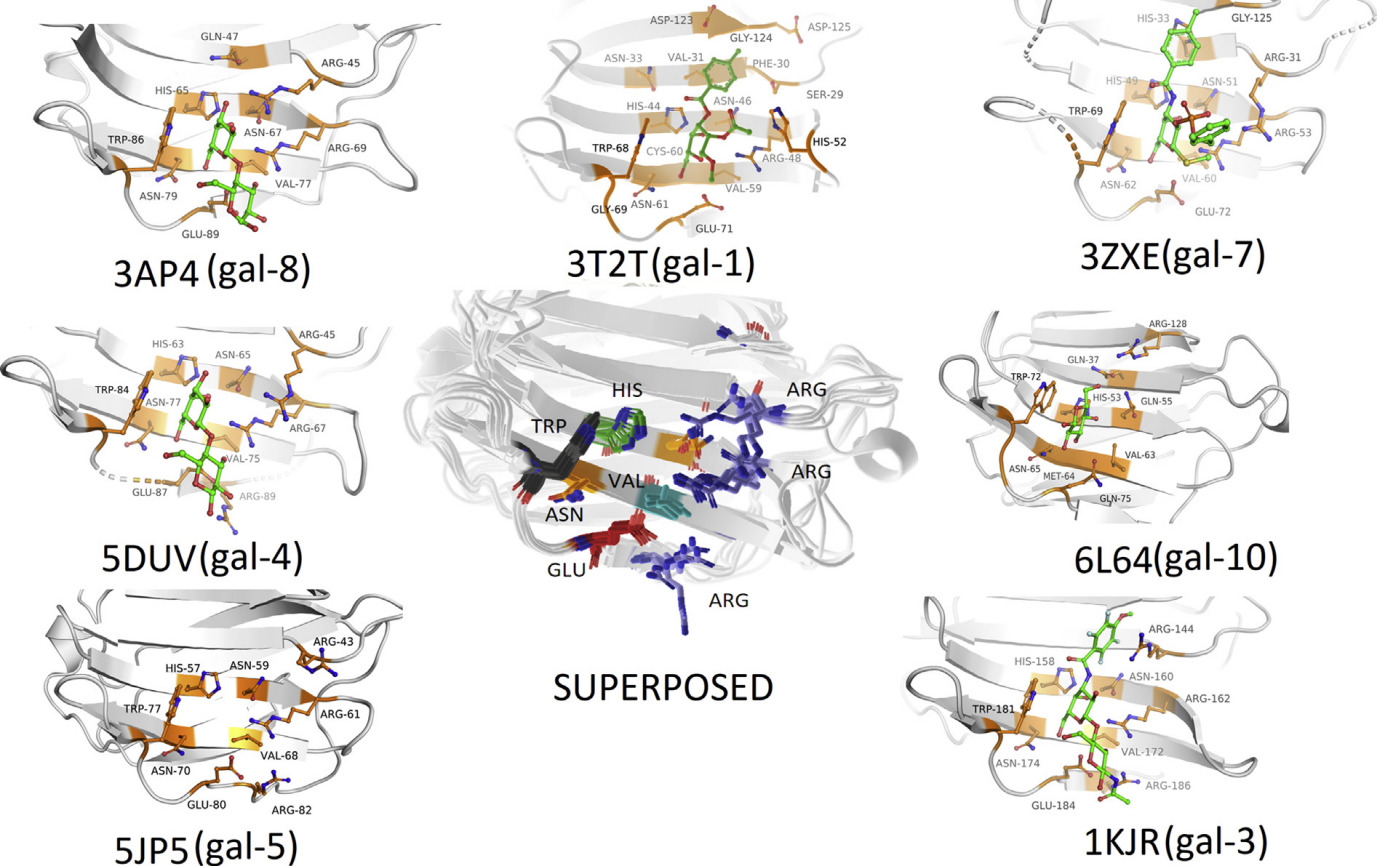
**A layout of a set of crystallographic poses of galectin family bound to its native ligand.** Also shown in the center is the superposed poses of all galectins.

The question arises: how representative are our observations made based on the simulation of galectin-3 with a prototypical ligand and its synthetic derivative? A comparison of experimentally solved crystal structures (PDB:3T2T-galectin-1, PDB:1KJR-galectin-3, PDB:5DUV-galectin-4, PDB:5JP5-galectin-5, PDB:3ZXE-galectin-7, PDB:3AP4-galectin-8, and PDB:6L64-galectin-10) provided insightful information, which further rationalizes our efforts toward finding the binding pathways for galectin-3. A comparison of galectin-3 with other galectin isoforms such as galectin-1, 10, 4, 5, 7, and 8 shows similar binding site chemistry exhibited by galectin-3 (see Fig. 10). Presence of tryptophan residue, which provides a hydrophobic anchor site and the strategically placed arginines along with presence of histidines and asparagine residues in the CRD of galectins, can be suitably utilized toward design of efficient therapeutic molecules, which can competitively inhibit this class of proteins. Our extensive binding simulation study in the present work therefore bolsters enrichment of contemporary information from a “bulk-to-site” perspective.

As an important result, the current simulations identified that the origin of superior binding affinity of the LacNAc derivative over its parent ligand is rooted in its longer ligand-residence time in the binding pocket. This was unequivocally demonstrated by an estimate of off-rate constants for both the ligands, which was 25 times slower in case of the synthetic derivative. The prediction of longer residence time as the crucial factor for the efficacy of synthetic ligand mirrors seminal hypothesis of Copeland *et al.* (14, 15). Although there are established experimental methods for measuring dissociative half-life (such as separation methods, spectroscopic differentiation, recovery of biological activity, and immobilized binding partner methods) (14), it is so far not possible to elucidate the chemical mechanism of longer/shorter attachment of receptor–ligand complexes through these rather expensive and time-consuming techniques, which in contrast are achievable through computational approaches such as metadynamics and steered molecular dynamics. Computationally, it is possible to get an estimate of the trends and chemistry behind a particular macromolecular recognition event provided enough sampling is accumulated in the backend.

In last few years, research led by Nilsson *et al.* has explored various different chemistries to target Galectin-3. One such effort reports precise investigation of the phenyltriazolyl-thiodigalactosides fluoro-interactions with galectin-3 generated compounds with reportedly high affinity (*K*_*d*_ 7.5 nM) and selectivity (46-fold) over galectin-1 for asymmetrical thiodigalactosides with one trifluorophenyltriazole and one coumaryl moiety (26). In another work where C1-galactopyranosyl heteroaryl derivatives were evaluated, it was observed that selectivity and affinity are driven by the structure of the aryl substituent to give compounds selective for either galectin-1 or galectin-3. The affinities from this work were found to be close to or better than those of lactose and other natural galectin-binding disaccharides, selectivities offered by the C1-heteroaryl groups are better than that of lactose and at the same time compound drug-like properties are potentially better than those of natural saccharides (27). In a separate work, a series of 3-(4-(2,3,5,6-tetrafluorophenyl)-1,2,3-triazol-1-yl)-thiogalactosides with different para substituents were evaluated against galectin-3 where it was noticed that inhibitors substituted at the 3-position of a thiodigalactoside core cause the formation of an aglycone-binding pocket through the displacement of an arginine residue (Arg144) from its position in the apoprotein. Another crucial observation from the authors was that for the other ligands, the affinity appeared to be regulated mainly by desolvation effects, disfavoring the polar substituents, but this theory is partly opposed by these class of compounds where the 2,3,5,6-tetrafluorophenyl functional group forms cation–pi interaction with Arg144, which stacks on top of the substituted tetrafluorophenyl group in all crystal structure complexes (28). Contemporarily, fluorine interactions were further investigated systematically using phenyltriazolyl-thiogalactosides fluorinated singly or multiply at various positions on the phenyl ring. Galectin-3 X-ray structures with these ligands revealed potential orthogonal fluorine–amide interactions with backbone amides and one with a side-chain amide. The two interactions involving main-chain amides have a strong influence on affinity, whereas the interaction with the side-chain amide did not influence affinity (29).

Finally, galectin-3 being a cancer drug target, the noncanonical binding regions of galectin-3, as reported in the current work, can be used to develop possible lead molecules that can target the sites, which in turn can reduce the binding affinity of galectin-3 to polysaccharides, since it has been proposed that binding on one side attenuates the binding affinity of the other side (22).

## Experimental procedures

The crystal structure of galectin-3 (PDB ID: 1KJL (12)) without the substrate serves as the initial structure of the protein for exploring binding events of the parent ligand LacNAc and LacNAC derivative. The ligand-free galectin-3 is placed at the center of the dodecahedron box with 11 Å distance between protein surface and box. The protein system is solvated with 15,644 TIP3P (30) water molecules and sufficient number of potassium and chloride ions were added to maintain the KCl concentration to 150 mM and render the system electroneutral. Subsequently, three copies of LacNAc molecules were introduced at random positions and orientations in the bulk solvent media of the system. The protein and ligand molecule were kept mutually far apart at the start of the simulations. Figure 3*A* represents a typical snapshot of the initial simulation setup adopted in the current work. Throughout the simulations, the ligand molecules were allowed to diffuse freely in the absence of any artificial bias. The system included a total of 49,441 atoms for the simulation of native ligand LacNAc.

An identical procedure was repeated for simulating the diffusion of LacNAc derivative around protein. In this case the system was constituted of the protein, three copies of “LacNAc derivative,” and 13,813 TIP3P water models, 150 mM KCl in a dodecahedron box of same volume as in the case of system with LacNAc, giving rise to a system size of 43,963 atoms. Amber14sb (31) force field was used for protein. The parameters for the LacNAc were generated from glycam carbohydrate builder while the parameters of LacNAc derivative were optimized using GAAMP.

All unbiased MD simulations were performed with Gromacs-2018.6 (or higher version) simulation package (32, 33) using leap-frog integrator with time step of 2 fs. The simulations were performed in NPT ensemble. Nose–Hoover thermostat (34, 35) and Parrinello–Rahman barostat (36) were used to maintain the average temperature of the system at 310.15 K with a relaxation time of 1.0 ps and at 1 bar constant pressure with a coupling constant of 5.0 ps respectively. The Verlet (37) cutoff scheme was employed in the simulations with the Lennard–Jones interactions being cut off at 1.2 nm. Particle Mesh Ewald (PME) summation (38, 39) was used to treat long-range electrostatic interactions. All bond lengths involving hydrogen atoms were constrained using the LINCS algorithm (40) for protein–ligand and SETTLE algorithm (41) for TIP3P water molecules. Multiple realizations of the unbiased MD simulations were initiated by assigning random velocities to all particles. The simulations were boosted by usage of Graphics Processing Units (GPUs) (42).

Multiple long independent and unbiased binding MD trajectories were generated, each of which ranged between 1 and 2.57 μs in case of LacNAc and its derivative. The simulations were terminated only after one of the copies of the ligand got bound in the CRD and remained settled there for the rest of the simulation period. The protein–ligand binding process was verified by visual inspection and by computing (i) the radial distance between respective center of mass of the binding pocket and the ligand and (ii) the RMSD of the simulated bound conformation from that of the X-ray crystal structure. The binding pocket was defined by a set of protein heavy atoms within 0.5 nm of ligand in the X-ray structure of bound conformation. The ligand was confirmed to be bound to galectin-3 when the RMSD remained below 0.2 nm and the cavity–ligand distance was below 0.5 nm for a simulation duration of at least 100 ns. Apart from the microsecond long trajectories, 100 short independent trajectories, each 100 ns long, were initiated from different ligand-bound states of system involving protein and LacNAc, which were *a priori* curated *via* k-means clustering of long binding trajectories.

The cumulative short and long trajectories were then aggregated to construct an MSM for quantitative description of recognition processes in case of both the ligands (16, 17, 18, 19) and for the identification of kinetically relevant states and their interconversion rates from the simulated trajectories. PyEMMA (43) (http://pyemma.org) was used to construct and analyze the MSM from all the obtained trajectories. Fig. S7 illustrates the MSM protocol employed in the current work for both the ligands. The nearest-neighbor binary contact matrix between heavy atoms of protein residues and ligand with a cutoff of 0.5 nm was used as input coordinates for MSM building. Time-lagged independent component analysis (tICA) (44, 45, 46) with a lag time of 10 ns was used for dimensionality reduction, which projected the high dimensional data onto 20 tICA components based on kinetic variance. This 20-dimensional tICA data was clustered into 500 clusters using k-means clustering algorithm (47). To get the appropriate lag time, 500-microstate MSMs were built at different lag times. The implied timescale (ITS) plot plateaued beyond 10 ns and hence a lag time of 10 ns was chosen to build the MSM, which ensures the Markovianity of the model (see Fig. S7 for both the ligands). For a comprehensible understanding of the ligand recognition process, the 500-microstate MSM was coarse-grained into a set of macro states. The timescale separation of ITS plot suggested the presence of four macrostates. Accordingly, a coarse-grained four-state kinetic model was constructed with a 10 ns lag-time. PCCA+ was used for coarse-graining purpose. The stationary population of the four macro states was computed.

For the other ligand, *i.e.*, “LacNAC derivative,” a similar protocol was employed, wherein a set of 36 short independent trajectories, each 250 ns long, were initiated. Similar protocol as the parent ligand has been followed in this ligand as well, where we computed the contact matrix between ligand-2 and amino-acid residues of the protein. The obtained 138 dimensional data is projected onto 20 dimensions using tICA for a lag time of 100 steps (1 ns). The projected data is subjected to clustering using k-means clustering algorithm and a similar number of microstates (*i.e.*, 500) were chosen in this case of LacNAc derivative as well. Based on ITS, a 3-macrostate MSM is built using the discretized data. The representative snapshots of the three states are shown in the figures. For LacNAC derivative, the ITS plateaued beyond 7.5 ns and hence a lag time of 7.5 ns was chosen to build the MSM.

Binding free energies (Δ*G*) of LacNAc and its synthetic derivative to galectin-3 were calculated from the stationary populations of bound and unbound macrostates as obtained from the MSM,

(1) $$\text{Δ}G=-RTlog\left(\frac{\pi_{bound}}{\pi_{unbound}}\right)$$

where *π*_*bound*_ and *π*_*unbound*_ represent stationary population of bound and unbound macrostates, R is the universal gas constant, and T is the absolute temperature. This result is converted to standard free energy Δ*G*^0^ for comparison with experimental data.

The kinetic parameters reported in the current article were calculated using the mean first passage time (MFPT) between MSM-derived key macrostates. The MFPT was computed as the average time taken for the transition from the initial(unbound) to the final(bound) macrostate. The calculation included both the direct transition from the initial state to the final state and transitions through other intermediate states. The on-rate and off-rate constants were respectively calculated as $k_{on}=\frac{1}{MFPT_{on}C}$ and $k_{off}=\frac{1}{MFPT_{off}}$, where *C* is the ligand concentration. Finally, transition path theory (TPT), proposed by Metzner *et al.* (21), was employed for enumerating all possible transition paths between unbound and bound macrostates and their respective fluxes were computed.

As would be illustrated in the Results section, a key finding of the MSM-based kinetic analysis is the considerably slow ligand-unbinding rate of the LacNAC derivative over LacNac, which is suggestive of their distinct residence times in the pocket. In a bid to characterize the key molecular determinants responsible for distinct ligand residence time, we simulate the ligand exit process from the pocket using metadynamics (48, 49). Based on visual inspection, we chose a combination of two collective variables (CVs) for biasing the metadynamics simulations: (i) distance between center of mass of ligand and residue 162 and (ii) number of hydrogen bonds between ligand and residues 158(his), 162(Arg), 174(Asn),184(Glu). A well-tempered variant of the metadynamics simulation (50) was employed for our study where a history-dependent bias *V*(*S*, *t*) is typically constructed in the form of periodically added repulsive Gaussians, where *S* is the chosen CV, which could be multidimensional. At any time given time t, the free energy *F*(*S*) can be obtained from the deposited bias *V*(*S*, *t*) as per the following equation:

$$V\left(S,t\to \infty \right)=-\frac{\Delta T}{T+\Delta T}F\left(S\right)+C$$

where *T* is the simulation temperature, which is 300K, and Δ*T* is the tempering factor through which the amplitude of the bias deposited at a point in the collective variable space is tuned down, and C(t) is a time-dependent constant, which is irrelevant for the present work. The history-dependent biases were added along both CVs at an interval of 500 steps. Other numerical parameters include the initial Gaussian hill height h = 1.2 kJ/mol and Gaussian width w = 0.00981 nm (distance), 0.227 (hydrogen bonds), and bias factor of 6.0. All metadynamics simulations were performed using Gromacs2018 and its PLUMED (51, 52) plugin.

The residue–ligand interactions are calculated with Arpeggio (http://biosig.unimelb.edu.au/arpeggioweb/) and rendered with Pymol (http://pymol.org). MMPBSA calculations were conducted with *g*_*mmpbsa*_ tool (53).

## Data availability

All data are present in the article. The simulation trajectories can be made available upon reasonable request to the authors.

## Supporting information

This article contains supporting information.

## Conflict of interest

The authors declare that they have no conflicts of interest with the contents of this article.

## References

[1] Sharon N. (2008). Lectins: Past, present and future1. Biochem. Soc. Trans..

[2] Sharon N., Lis H. (2004). History of lectins: From hemagglutinins to biological recognition molecules. Glycobiology.

[3] Houzelstein D., Gonclalves I.R., Fadden A.J., Sidhu S.S., Cooper D.N.W., Drickamer K., Leffler H., Poirier F. (2004). Phylogenetic analysis of the vertebrate galectin family. Mol. Biol. Evol..

[4] Goetz J.G., Joshi B., Lajoie P., Strugnell S.S., Scudamore T., Kojic L.D., Nabi I.R. (2008). Concerted regulation of focal adhesion dynamics by galectin-3 and tyrosine-phosphorylated caveolin-1. J. Cell Biol..

[5] Barondes S.H., Cooper D.N., Gitt M.A., Leffler H., Galectins (1994). Structure and function of a large family of animal lectins. J. Biol. Chem..

[6] ñberg C.T., Blanchard H., Leffler H., Nilsson U.J. (2008). Protein subtype-targeting through ligand epimerization: Talose-selectivity of galectin-4 and galectin-8. Bioorg. Med. Chem. Lett..

[7] Nabi I.R., Shankar J., Dennis J.W. (2015). The galectin lattice at a glance. J. Cell Sci..

[8] Garner O., Baum L. (2008). Galectin-glycan lattices regulate cell-surface glycoprotein organization and signalling. Biochem. Soc. Trans..

[9] Guha P., Kaptan E., Bandyopadhyaya G., Kaczanowska S., Davila E., Thompson K., Martin S.S., Kalvakolanu D.V., Vasta G.R., Ahmed H. (2013). Cod glycopeptide with picomolar affinity to galectin-3 suppresses T-cell apoptosis and prostate cancer metastasis. Proc. Natl. Acad. Sci. U. S. A..

[10] Braeuer R.R., Zigler M., Kamiya T., Dobroff A.S., Huang L., Choi W., McConkey D.J., Shoshan E., Mobley A.K., Song R., Raz A., Bar-Eli M. (2012). Galectin-3 contributes to melanoma growth and metastasis via regulation of NFAT1 and autotaxin. Cancer Res..

[11] Blanchard H., Yu X., Collins P.M., Bum-Erdene K. (2014). Galectin-3 inhibitors: A patent review (2008-present). Expert Opin. Ther. Pat..

[12] Sarme P., Arnoux P., Kahl-Knutsson B., Leffler H., Rini J.M., Nilsson U.J. (2005). Structural and thermodynamic studies on cation-pi interactions in lectin-ligand complexes: High-affinity galectin-3 inhibitors through fine-tuning of an arginine-arene interaction. J. Am. Chem. Soc..

[13] Seetharaman J., Kanigsberg A., Slaaby R., Leffler H., Barondes S.H., Rini J.M. (1998). X-ray crystal structure of the human galectin-3 carbohydrate recognition domain at 2.1-A resolution. J. Biol. Chem..

[14] Copeland R., Pompliano D., Meek T. (2006). Drug-target residence time and its implications for lead optimization. Nat. Rev. Drug Discov..

[15] Tummino P.J., Copeland R.A. (2008). Residence time of receptor? Ligand complexes and its effect on biological function. Biochemistry.

[16] Chodera J.D., Noe F. (2014). Markov state models of bimolecular conformational dynamics. Curr. Opin. Struct. Biol..

[17] Noe F., Horenko I., Schutte C., Smith J.C. (2007). Hierarchical analysis of conformational dynamics in biomolecules: Transition networks of metastable states. J. Chem. Phys..

[18] Bowman G.R., Pande V.S., Noe F. (2014).

[19] Plattner N., Noe F. (2015). Protein conformational plasticity and complex ligand-binding kinetics explored by atomistic simulations and Markov models. Nat. Commun..

[20] Meynier C., Feracci M., Espeli M., Chaspoul F., Gallice P., Schiff C., Guerlesquin F., Roche P. (2009). NMR and MD investigations of human galectin-1/oligosaccharide complexes. Biophys. J..

[21] Metzner P., Schootte C., Vanden-Eijnden E. (2009). Transition path theory for Markov jump processes. Multiscale Model. Simul..

[22] Miller M.C., Ippel H., Suylen D., Klyosov A.A., Traber P.G., Hackeng T., Mayo K.H. (2016). Binding of polysaccharides to human galectin-3 at a noncanonical site in its carbohydrate recognition domain. Glycobiology.

[23] Mondal J., Ahalawat N., Pandit S., Kay L.E., Vallurupalli P. (2018). Atomic resolution mechanism of ligand binding to a solvent inaccessible cavity in T4 lysozyme. PLoS Comput. Biol..

[24] Ahalawat N., Mondal J. (2018). Mapping the substrate recognition pathway in cytochrome P450. J. Am. Chem. Soc..

[25] Maschera B., Darby G., Palú G., Wright L.L., Tisdale M., Myers R., Blair E.D., Furfine E.S. (1996). Human immunodeficiency virus: Mutations in the viral protease that confer resistance to saquinavir increase the dissociation rate constant of the protease-saquinavir complex. J. Biol. Chem..

[26] Peterson K., Kumar R., Stenstrom O., Verma P., Verma P.R., Haykansson M., Kahl-Knutsson B., Zetterberg F., Leffler H., Akke M., Logan D.T., Nilsson U.J. (2018). Systematic tuning of fluoro-galectin-3 interactions provides thiodigalactoside derivatives with single-digit nM affinity and high selectivity. J. Med. Chem..

[27] Dahlqvist A., Leffler H., Nilsson U.J. (2019). C1-galactopyranosyl heterocycle structure guides selectivity: Triazoles prefer galectin-1 and oxazoles prefer galectin-3. ACS Omega.

[28] Kumar R., Peterson K., Misini Ignjatoviaá M., Leffler H., Ryde U., Nilsson U.J., Logan D.T. (2019). Substituted polyfluoroaryl interactions with an arginine side chain in galectin-3 are governed by steric-, desolvation and electronic conjugation effects. Org. Biomol. Chem..

[29] Kumar R., Ignjatovic M.M., Peterson K., Olsson M., Leffler H., Ryde U., Nilsson U.J., Logan D.T. (2019). Structure and energetics of ligand-fluorine interactions with galectin-3 backbone and side-chain amides: Insight into solvation effects and multipolar interactions. ChemMedChem.

[30] Jorgensen W.L., Chandrasekhar J., Madura J.D., Impey R.W., Klein M.L. (1983). Comparison of simple potential functions for simulating liquid water. J. Chem. Phys..

[31] Maier J.A., Martinez C., Kasavajhala K., Wickstrom L., Hauser K.E., Simmerling C. (2015). ff14SB: Improving the accuracy of protein side chain and backbone parameters from ff99SB. J. Chem. Theory Comput..

[32] Berendsen H., van der Spoel D., van Drunen R. (1995). GROMACS: A message-passing parallel molecular dynamics implementation. Comput. Phys. Commun..

[33] Abraham M.J., Murtola T., Schulz R., Paill S., Smith J.C., Hess B., Lindahl E. (2015). GROMACS: High performance molecular simulations through multi-level parallelism from laptops to supercomputers. SoftwareX.

[34] Nose S. (1984). A molecular dynamics method for simulations in the canonical ensemble. Mol. Phys..

[35] Hoover W. (1985). Canonical dynamics: Equilibrium phase-space distributions. Phys. Rev. A.

[36] Parrinello M., Rahman A. (1981). Polymorphic transitions in single crystals: A new molecular dynamics method. J. Appl. Phys..

[37] Pail S., Hess B. (2013). A flexible algorithm for calculating pair interactions on {SIMD} architectures. Comput. Phys. Commun..

[38] Darden T., York D., Pederson L.G. (1993). Particle mesh Ewald: An *N*⋅log(*N*) method for Ewald sums in large systems. J. Chem. Phys..

[39] Essman U., Perera L., Berkowitz M.L., Darden T., Lee H., Pederson L.G. (1995). A smooth particle mesh Ewald method. J. Chem. Phys..

[40] Hess B., Bekker H., Berendsen H.J.C., Fraaije J.G.E.M. (1997). LINCS: A linear constraint solver for molecular simulations. J. Comput. Chem..

[41] Miyamoto S., Kollman P. (1992). Settle: An analytical version of the SHAKE and RATTLE algorithm for rigid water models. J. Comput. Chem..

[42] Kutzner C., Paill S., Fechner M., Esztermann A., de Groot B.L., Grubmuller H. (2015). Best bang for your buck: GPU nodes for GROMACS biomolecular simulations. J. Comput. Chem..

[43] Scherer M.K., Trendelkamp-Schroer B., Paul F., Perez-Hernandez G., Hoffmann M., Plattner N., Wehmeyer C., Prinz J.-H., Noe F. (2015). PyEMMA 2: A software package for estimation, validation, and analysis of Markov models. J. Chem. Theory Comput..

[44] Molgedey L., Schuster H.G. (1994). Separation of a mixture of independent signals using time delayed correlations. Phys. Rev. Lett..

[45] Parez-Hernandez G., Paul F., Giorgino T., De Fabritiis G., Noe F. (2013). Identification of slow molecular order parameters for Markov model construction. J. Chem. Phys..

[46] Schwantes C.R., Pande V.S. (2013). Improvements in Markov state model construction reveal many non-native interactions in the folding of NTL9. J. Chem. Theory Comput..

[47] Lloyd S. (2006). Least squares quantization in PCM. IEEE Trans. Inf. Theory.

[48] Laio A., Parrinello M. (2002). Escaping free energy minima. J. Chem. Phys..

[49] Bussi G., Laio A. (2020). Using metadynamics to explore complex free-energy landscapes. Nat. Rev. Phys..

[50] Barducci A., Bussi G., Parrinello M. (2008). Well-tempered metadynamics: A smoothly converging and tunable free-energy method. Phys. Rev. Lett..

[51] Bonomi M., Branduardi D., Bussi G., Camilloni C., Parrinello M. (2009). PLUMED: A portable plugin for free-energy calculations with molecular dynamics. Comput. Phys. Commun..

[52] Tribello G.A., Bonomi M., Branduardi D., Camilloni C., Bussi G. (2014). {PLUMED} 2: New feathers for an old bird. Comput. Phys. Commun..

[53] Kumari R., Kumar R., Lynn A. (2014). g_mmpbsa: A GROMACS tool for high-throughput MM-PBSA calculations. J. Chem. Inf. Model..

