# Supplementary material for: Molecular dynamics simulations elucidate oligosaccharide recognition pathways by galectin-3 at atomic resolution
Source: J Biol Chem. 2021 Oct 5;297(5):101271. doi: 10.1016/j.jbc.2021.101271 (PMC8571523; doi:10.1016/j.jbc.2021.101271)
Supplement: Supplemental Figures S1–S7 [file mmc1.pdf]

Supporting Information for "Molecular Dynamics Simulations Elucidate Oligosaccharide Recognition Pathways by Galectin-3 at Atomic Resolution"

Jaya Krishna Koneru, Suman Sinha,* and Jagannath Mondal*

Tata Institute of Fundamental Research, Center for Interdisciplinary sciences, Hyderabad 500046, India

E-mail: ssinha@tifrh.res.in; jmondal@tifrh.res.in,+914020203091

Description of Supplemental movie:

Movie S1 demonstrates a representative Molecular dynamics simulation trajectory capturing the process of ligand N-acetyllactosamine (LacNAc) binding to Galectin-3 in atomistic resolution.

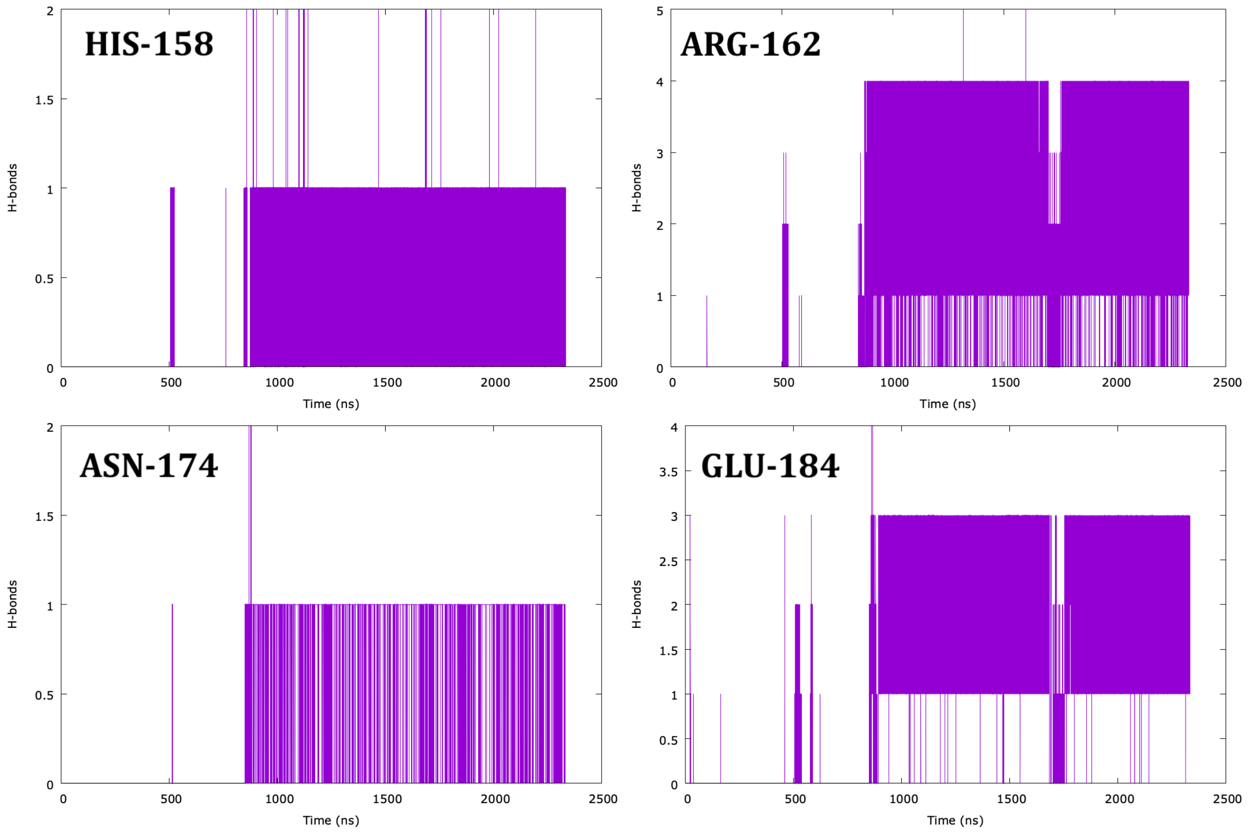

Figure S1: Time profile of hydrogen bonds between the LacNac and key amino acid residues around binding site. Presence of persistent hydrogen bonds between LacNac and key amino acid residues in the binding pocket is evident beyond the binding event.

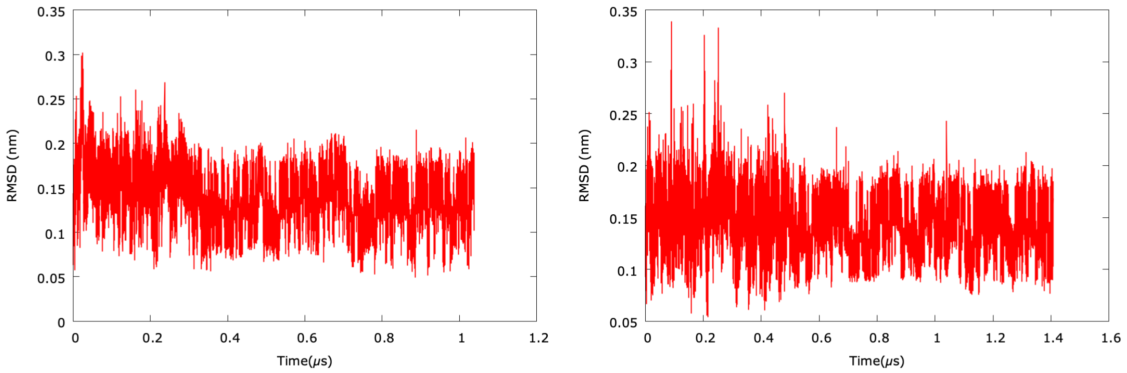

Figure S2: RMSD profile of binding pocket of galectin-3 CRD with respect to crystal structure (pdb id: 1KJL). Both representative trajectories indicate no significant change in pocket before or after ligand binding

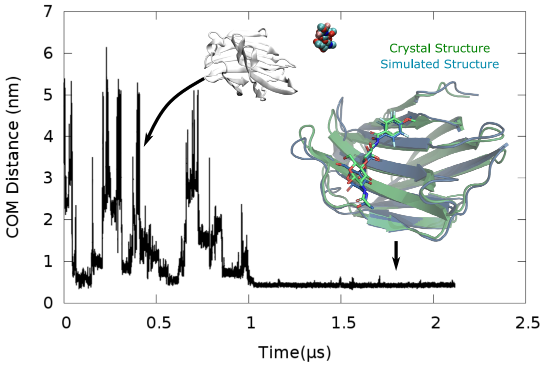

Figure S3: The time profile of binding trajectory for LacNAc-derivative.

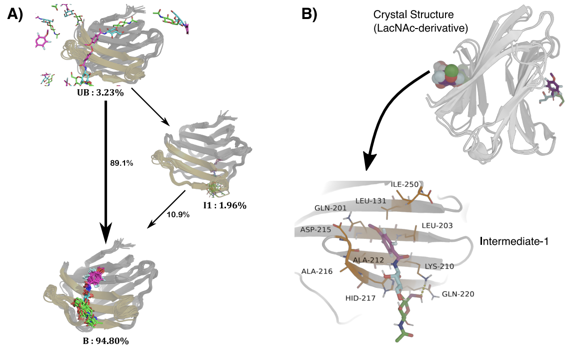

Figure S4: Network showing the LacNAc-derivative's binding pathway. Path flux is represented with thickness of arrows and path percentages are indicated. B. Key interactions stabilising the non-native encounter-complex of Galectin-3/LacNAc-derivative.

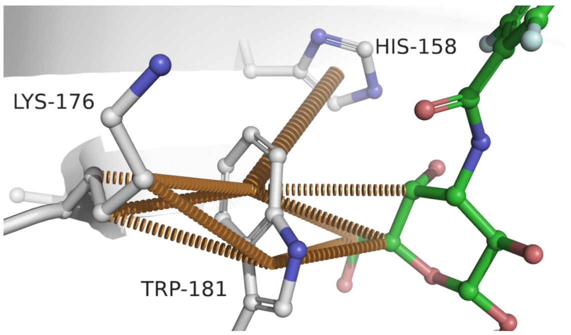

Figure S5: Network of Trp181 stabilization.

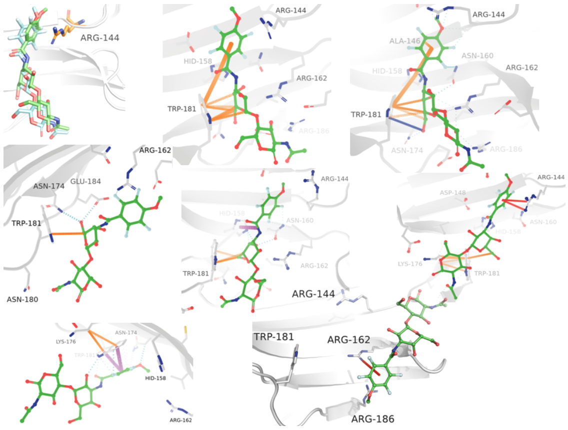

Figure S6: Details of Key residue-interactions with LacNAc-derivative in the binding pocket

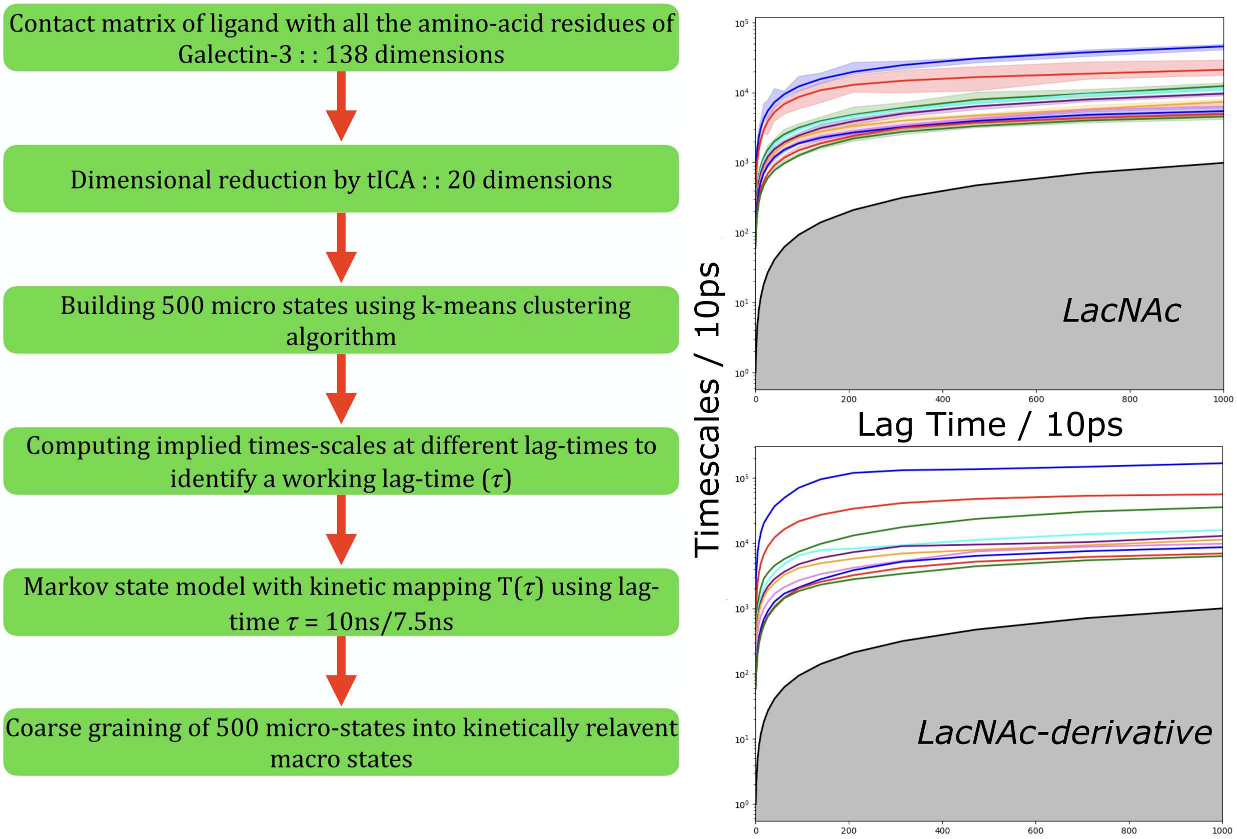

Figure S7: The outline of MSM protocol adopted in the current article. Also shown is the plot of implied time scale as a function of lag-times
